# Sialorrhoea and Ptyalomania

**Published:** 1914-05

**Authors:** 


Sialorrhoea and Ptyalomania. Maurice Loeper of Paris, *Le Progres Med.,* Nov. 27, 1913, defines the latter as a voluntary if not conscious tic, occurring in the nervous and also in certain gastropaths, consisting in a spitting habit without excess of saliva. Sialorrhœa, though not definitely limited to certain diseases, is especially noted in ulcerative lesions and particularly those of the cardia and the lesser curvature. The quantity of saliva is often difficult to estimate on account of being swallowed, but it may amount to 700, or even 1,500 c.c. a day. The specific gravity (1,004) and mineral constituents do not differ materially from the normal.

